# Supplementary material for: Aorta-specific DNA methylation patterns in cell-free DNA from patients with bicuspid aortic valve-associated aortopathy
Source: Clin Epigenetics. 2021 Jul 28;13:147. doi: 10.1186/s13148-021-01137-y (PMC8320174; doi:10.1186/s13148-021-01137-y)
Supplement: Supplementary file 1 — Additional file 1. Supplementary Material. [file 13148_2021_1137_MOESM1_ESM.docx]

**Supplementary Material**

**Table of contents**

Supplemental Methods page 2

Table S1 page 8

Table S2 page 10

Table S3 page 11

Table S4 page 12

Table S5 page 13

Fig. S1 page 14

Fig. S2 page 15

Fig. S3 page 16

Fig. S4 page 17

**Supplemental Methods**

**Analysis of myocardial apoptosis**

A green fluorescent signal was observed at 488 nm in regions that contained apoptotic or necrotic myocardial cells while DAPI-stained nuclei emitted a blue signal at 405 nm. Images were analyzed using ImageJ and the Fiji plugin. The brightness and contract were adjusted to a minimum intensity of 122 arbitrary units (a.u.) and a maximum intensity of 145 a.u. for each image taken at 488 nm while all images taken at 405 nm were adjusted to a minimum intensity of 105 and a maximum intensity of 145. This allowed for the clearest visualization of the TUNEL and DAPI signals within each set of images as these settings removed any background staining within the images. Then, using the selection tool, the image of the tissue section taken at 488 nm was traced and edges and any areas of thin or torn tissue were omitted. This ensured that any cells that stained positively for TUNEL and DAPI due to physical damage, rather than immune-mediated damage, did not skew the results. This traced outline was then pasted and aligned with the image taken at 405 nm which contained all of the DAPI-stained cells. The traced outlined was applied, which removed all areas of the image outside of the outline, leaving behind only the tissue section of interest. Next, both images taken at 488 nm and 405 nm were further enhanced by removing any residual noise using the “despeckle” option in Fiji. Following this, the cropped image taken at 405 nm was then used to create a mask, which served as a reference for the number and location of each nucleated cells within the tissue section of interest when determining the colocalization of TUNEL and DAPI. Finally, the “coloc 2” test in Fiji was employed. Channel 1 was assigned as the image taken at 488 nm and displayed all TUNEL-positive cells and channel 2 was assigned as the image taken at 405 nm and displayed all DAPI-positive cells. The mask created from the image taken at 405 nm was also plugged in. The “coloc 2” test was allowed to run and output listed the proportion of cells that displayed a colocalization of TUNEL and DAPI and, therefore, represented apoptotic or necrotic cells within the tissue section.

***In silico* identification of candidate ventricle-specific DMRs**

**Converting file formats**

*BigWig to Wig*: $ bigWigToWig <file_name.bw> <file_name.wig>

*Wig to Bed*: $ convert2bed -i wig <file_name.wig> <file_name.bed>

*Bed to Bedgraph*: $ awk '{ print $1"\t"$2"\t"$3"\t”$5 }’ file_name.bed > file_name.bedgraph

*Sorted Bedgraph*: $ sortBed -i file_name.bedgraph > file_name_sorted.bedgraph

**Creating input files for analysis in metilene**

**Comparing ventricular methylomes to non-ventricular tissue methylomes**

$ metilene_input.pl -in1 GSM1010978_Left_Ventricle.sorted,GSM1010988_Right_Ventricle.sorted,GSM983650_Left_Ventricle2.sorted -in2 GSM1010979_Thymus.sorted,GSM1010980_Ovary.sorted,GSM1010981_Adrenal_Gland.sorted,GSM1010983_Adipose_Tissue.sorted,GSM1010984_Gastric.sorted,GSM1010986_Psoas_Muscle.sorted,GSM1010987_Right_Atrium.sorted,GSM1010989_Sigmoid_Colon.sorted,GSM1112838_Brain_Hippocampus_Middle.sorted,GSM1127054_Breast_Myoepithelial_Cells.sorted,GSM916049_Adult_Liver.sorted,GSM916050_Brain_Hippocampus_Middle2.sorted,GSM983645_Sigmoid_Colon2.sorted,GSM983646_Small_Intestine.sorted,GSM983647_Lung.sorted,GSM983648_Aorta.sorted,GSM983649_Esophagus.sorted,GSM983651_Pancreas.sorted,GSM983652_Spleen.sorted -h1 Ventricles -h2 Tissues -out Metilene_Ventricles_Tissues.input & > Metilene_Ventricles_Tissues.input

**General Script:**

$ metilene_input.pl -in1 <comma separated sorted bedgraph files of epigenomes from left and right ventricle tissues> -in2 <comma separated sorted bedgraph files of epigenomes from non-ventricular tissues> -h1 Ventricles -h2 Tissues/Cells -out Metilene_Filename.input

**Comparing ventricular methylomes to hematopoietic cell methylomes**

$ metilene_input.pl -in1 GSM1010978_Left_Ventricle.sorted,GSM1010988_Right_Ventricle.sorted,GSM983650_Left_Ventricle2.sorted -in2 EGAX00001086969_Neutrophil_venous.sorted,EGAX00001086971_Neutrophil_venous.sorted,EGAX00001086972_Neutrophil_venous.sorted,EGAX00001097771_neutrophil_cord.sorted,EGAX00001097776_Neutrophil_venous.sorted,EGAX00001128259_plasma_cell_bone.sorted,EGAX00001147725_macrophage_venous.sorted,EGAX00001195936_memory_B_venous.sorted,EGAX00001208464_erythroblast_cord.sorted,EGAX00001208466_CD4_alpha_beta_T_cell_venous.sorted,EGAX00001236255_inflammatory_macrophage_cord.sorted,EGAX00001236257_regulatory_T_cell_venous.sorted,EGAX00001236260_eosinophil_venous.sorted,GSM916052_CD34_Primary_Cells.sorted -h1 Ventricles -h2 Cells -out Metilene_Ventricles_Cells.input & > Metilene_Ventricles_Cells.input

**General Script:**

$ metilene_input.pl -in1 <comma separated sorted bedgraph files of epigenomes from left and right ventricle tissues> -in2 <comma separated sorted bedgraph files of epigenomes from hematopoietic cells> -h1 Ventricles -h2 Tissues/Cells -out Metilene_Filename.input

**Filtering and sorting DMRs by 10% to obtain an output file**

**Filtering and sorting DMRs Between ventricular and non-ventricular tissues**

$ metilene -M 25 -m 4 -d 0.1 -t 4 -f 1 -a Ventricles -b Tissues -X 1 -Y 1 -v 0.7 Metilene_Ventricles_Tissues.input  > Metilene_Ventricles_Tissues.output | sort -V -k1,1 -k2,2n

**Filtering and sorting DMRs between ventricular tissues and hematopoietic cells**

$ metilene -M 25 -m 4 -d 0.1 -t 4 -f 1 -a Ventricles -b Tissues -X 1 -Y 1 -v 0.7 Metilene_Ventricles_Cells.input > Metilene_Ventricles_Cells.output | sort -V -k1,1 -k2,2n

**Filtering the output file to obtain DMRs with a methylation difference of 50-80%**

**Filtering DMRs between ventricular and non-ventricular tissues (difference of 50%)**

$ metilene_output.pl –q Metilene_Ventricles_Tissues.output –o Metilene_Ventricles_Tissues_Filtered –p 0.05 –d 0.5 –c 4 –l 0 -a Ventricles –b Tissues

**Filtering DMRs between ventricular tissues and hematopoietic cells (difference of 80%)**

$ metilene_output.pl –q Metilene_Ventricles_Cells.output –o Metilene_Ventricles_Cells_Filtered –p 0.05 –d 0.8 –c 4 –l 0 -a Ventricles –b Cells

**Finding common DMRs between the non-ventricular tissues and hematopoietic cells**

$ bedtools intersect –a < Metilene_Ventricles_Tissues_Filtered.bedgraph> -b < Metilene_Ventricles_Cells_Filtered.bedgraph>

**Primer annealing temperature optimization**

In order to determine the appropriate temperature that would allow for optimal primer annealing, a temperature gradient experiment was conducted. Each 200 μL PCR tube contained a total of 25 μL of reaction mixture. This mixture was comprised of 5 μL of 5X EpiMark® Hot Start Taq Reaction Buffer (New England BioLabs), 0.5 μL of 10 mM dNTP mix (Invitrogen), 0.5 μL of 10 μM forward primer, 0.5 μL of 10 μM reverse primer, 1.0 uL of 10 ng/μL bisulfite converted control human DNA (Qiagen), 0.125 μL of 5.00 U/mL EpiMark Hot Start Taq DNA Polymerase (New England BioLabs), and 17.38 μL of RNAse-free water to ensure the total volume was 25 uL. It should be noted that a master mix for each set of primers for a given DMR was created to reduce errors associated with pipetting volumes into each individual tube. Furthermore, the reagents were added in the order mentioned above. The tubes were then placed in the thermocycler and the PCR reaction was allowed to take place as follows: 3 minutes at 94ºC, 45 seconds at 94ºC, 45 seconds at one of eight temperatures between 55ºC - 65ºC, 1.5 minutes at 68ºC, steps 2-4 were repeated 40X, 10 minutes at 68ºC followed by an infinite hold at 4ºC. Products were visualized on a 3% agarose TAE gel using SYBR Green dye (Invitrogen) and a BioRad ChemiDoc Gel Imaging System.

**PCR of bisulfite-converted DNA**

Each 200 μL PCR tube contained 5 μL of 5X EpiMark Hot Start Taq Reaction Buffer (New England BioLabs), 0.5 μL of 10 mM dNTP mix (Invitrogen), 0.5 μL of 10 μM forward primer, 0.5 μL of 10 μM reverse primer, 1.0 μL of 10 ng/μL bisulfite-converted control human DNA (Qiagen), 0.125 μL of 5 U/mL EpiMark Hot Start Taq DNA Polymerase (New England BioLabs), and 17.38 μL of RNAse-free water for a final total volume of 25 μL. Tubes were then vortexed and centrifuged then placed in the thermocycler with the following program: 3 minutes at 94ºC, 45 seconds at 94ºC, 45 seconds at the determined Tm of the given primer, 1.5 minutes at 68ºC, steps 2-4 were repeated 40X, 10 minutes at 68ºC followed by an infinite hold at 4ºC.

**Table S1** Publicly-available methylomes of both ventricular and non-ventricular tissues and hematopoietic cells used for *in silico* DMR identification.

| **Tissue/cell type** | **Project** | **Accession number** |
| --- | --- | --- |
| Left ventricle | Roadmap | GSM1010978 |
| Left ventricle | Roadmap | GSM983650 |
| Right ventricle | Roadmap | GSM1010988 |
| Adipose tissue | Roadmap | GSM1010983 |
| Adrenal gland | Roadmap | GSM1010981 |
| Aorta | Roadmap | GSM983648 |
| Breast myoepithelial cells | Roadmap | GSM1127054 |
| Esophagus | Roadmap | GSM983649 |
| Gastric tissue | Roadmap | GSM1010984 |
| Hippocampus | Roadmap | GSM916050 |
| Hippocampus | Roadmap | GSM1112838 |
| Liver | Roadmap | GSM916049 |
| Lung | Roadmap | GSM983647 |
| Ovary | Roadmap | GSM1010980 |
| Pancreas | Roadmap | GSM983651 |
| Psoas muscle | Roadmap | GSM1010986 |
| Right atrium | Roadmap | GSM1010987 |
| Sigmoid colon | Roadmap | GSM983645 |
| Sigmoid colon | Roadmap | GSM1010989 |
| Small intestine | Roadmap | GSM983646 |
| Spleen | Roadmap | GSM983652 |
| Thymus | Roadmap | GSM1010979 |
| CD34+ cells | Roadmap | GSM916052 |
| Plasma cells (bone marrow) | Blueprint | EGAX00001128259 |
| Erythroblasts | Blueprint | EGAX00001208464 |
| Inflammatory macrophages | Blueprint | EGAX00001236255 |
| Neutrophils (cord blood) | Blueprint | EGAX00001097771 |
| Eosinophils | Blueprint | EGAX00001236260 |
| Macrophages (venous blood) | Blueprint | EGAX00001147725 |
| Memory B cells | Blueprint | EGAX00001195936 |
| Neutrophils (venous blood) | Blueprint | EGAX00001086969 |
| Neutrophils (venous blood) | Blueprint | EGAX00001086971 |
| Neutrophils (venous blood) | Blueprint | EGAX00001086972 |
| Neutrophils (venous blood) | Blueprint | EGAX00001097776 |
| Regulatory T cells (venous blood) | Blueprint | EGAX00001236257 |
| CD4+ cells (venous blood) | Blueprint | EGAX00001208466 |

**Table S2**  Putative aorta-specific DMRs identified including position, length in base pairs, number of CpG sites within each DMR and mean methylation differences as a percentage compared to non-ventricle tissues and hematopoietic cells.

| DMR | | | |  | Mean Methylation Difference (%) | |
| --- | --- | --- | --- | --- | --- | --- |
| Chr | **Start Position** | **End Position** | **Length (bp)** | **#CpGs** | **Non-aortic tissue** | **Hematopoietic cells** |
| 1* | 3,192,823 | 3,192,903 | 80 | 7 | -71.28 | -90.77 |
| 1 | 3,459,874 | 3,459,985 | 111 | 11 | -65.30 | -91.19 |
| 1 | 230,346,868 | 230,346,973 | 105 | 10 | -79.54 | -94.14 |
| 2 | 10,544,979 | 10,545,106 | 127 | 9 | -62.71 | -90.82 |
| 2* | 128,431,046 | 128,431,099 | 53 | 6 | -72.51 | -92.80 |
| 2 | 241,536,130 | 241,536,203 | 73 | 6 | -61.47 | -94.05 |
| 6 | 157,470,003 | 157,470,151 | 148 | 11 | -64.82 | -90.59 |
| 7 | 137,654,127 | 137,654,186 | 59 | 6 | -92.32 | -96.86 |
| 8 | 1,765,610 | 1,765,690 | 80 | 7 | 63.22 | 91.15 |
| 8* | 6,398,313 | 6,398,394 | 81 | 7 | -77.97 | -92.61 |
| 9 | 84,228,330 | 84,228,393 | 63 | 8 | -60.60 | -91.23 |
| 9* | 134,550,721 | 134,550,791 | 70 | 7 | -73.42 | -91.20 |
| 10 | 12,527,035 | 12,527,132 | 97 | 7 | -72.58 | -92.33 |
| 11** | **3,168,734** | **3,168,832** | **98** | **6** | **-70.60** | **-92.75** |
| 11 | 80,410,507 | 80,410,580 | 73 | 6 | -69.75 | -90.46 |
| 11 | 111,784,373 | 111,784,468 | 95 | 10 | -63.40 | -93.97 |
| 15* | 28,355,445 | 28,355,503 | 58 | 7 | -81.67 | -91.54 |
| 17 | 925,451 | 925,558 | 107 | 7 | -68.91 | -91.78 |
| 17 | 76,858,242 | 76,858,303 | 61 | 6 | -61.25 | -93.76 |
| 18** | **74,171,459** | **74,171,505** | **46** | **8** | **-74.26** | **-91.38** |
| 19* | 3,603,393 | 3,603,454 | 61 | 7 | -62.70 | -92.308 |
| 20** | **45,860,401** | **45,860,466** | **65** | **6** | **-86.70** | **-81.08** |
| 22** | **27,999,645** | **27,999,717** | **72** | **7** | **-75.36** | **-91.27** |
| 22 | 47,075,770 | 47,075,849 | 79 | 8 | -63.22 | -90.38 |

* These DMRs were selected for the first round of primer generation and testing.

** After primer generation and testing, these four DMRs were selected for complete validation.

**Table S3** Forward and reverse bisulfite PCR primer sequences designed using MethPrimer for the DMRs and the associated melting temperatures.

| **Chr** | **Amplimer size (bp)** | **Forward primer** | **Reverse primer** | **Tm (°C)** |
| --- | --- | --- | --- | --- |
| 11 | 127 | GGGTATTTAGTTATGAG-GGAATAATG | CAAACCTATCTTTAATTT-CCACCC | 55.7 |
| 18 | 120 | AGTTTAGGATTTGTGTT-ATTTAGGA | TAAAAAATATTACTATTA-ACATCATAACAA | 55 |
| 20 | 139 | GGAGTAAAATGAATAA-AATTTTTGTTGAGA | AAAAAACAAATACAAAA-AAACTACAAACC | 58.5 |
| 22 | 122 | GTTGAGGAATTGGAGG-AAAATTAA | ACAAACTACTAAACAAA-AAACACAA | 56.9 |

**Table S4** Correlation between aortic cfDNA levels for our candidate DMRs and elastin properties within aortic regions of elevated wall shear stress.

| DMR | Elastin Area | | Elastin Fibre Thickness | | Inter-fibre Distance | |
| --- | --- | --- | --- | --- | --- | --- |
|  | **R^2^** | ***P*** | **R^2^** | ***P*** | **R^2^** | ***P*** |
| Chr 11 | 0.0006 | 0.95 | 0.18 | 0.17 | 0.28 | 0.064 |
| Chr 18 | 0.01 | 0.82 | 0.27 | 0.19 | 0.091 | 0.32 |
| Chr 20 | 0.21 | 0.37 | 0.23 | 0.34 | 0.017 | 0.7 |
| Chr 22 | 0025 | 0.66 | 0.19 | 0.15 | 0.26 | 0.074 |

**Table S5** Correlation between aortic cfDNA levels for our candidate DMRs and protein levels within aortic regions of elevated wall shear stress.

| DMR | MMP-1 | | MMP-2 | | MMP-3 | | TGFβ-1 | | TIMP-1 | |
| --- | --- | --- | --- | --- | --- | --- | --- | --- | --- | --- |
|  | **R^2^** | ***P*** | **R^2^** | ***P*** | **R^2^** | ***P*** | **R^2^** | ***P*** | **R^2^** | ***P*** |
| 11 | 0.064 | 0.45 | 0.04 | 0.53 | 0.13 | 0.23 | 0.001 | 0.91 | 0.17 | 0.18 |
| 18 | 0.11 | 0.43 | 0.063 | 0.55 | 0.12 | 0.36 | 0.034 | 0.64 | 0.13 | 0.35 |
| 20 | 0.27 | 0.37 | 0.0022 | 0.92 | 0.14 | 0.41 | 0.15 | 0.39 | 0.0002 | 0.98 |
| 22 | 0.041 | 0.55 | 0.003 | 0.87 | 0.14 | 0.2 | 0.005 | 0.81 | 0.11 | 0.29 |

**Fig. S1**  (A) Total and aorta-specific cfDNA (as measured by the Chr 11 DMR) for patients with varying maximal aortic diameters. (B) Total and aorta-specific cfDNA (as measured by the Chr 18 DMR) for patients with varying maximal aortic diameters. (C) Total and aorta-specific cfDNA (as measured by the Chr 20 DMR) for patients with varying maximal aortic diameters. (D) Total and aorta-specific cfDNA (as measured by the Chr 22 DMR) for patients with varying maximal aortic diameters.

**Fig. S2** (A) Aorta tissue and DAPI staining (blue dots indicating stained cell nuclei). (B) Aorta tissue and TUNEL staining (green dots and arrows). (C) Colocalization of DAPI and TUNEL staining indicating the percentage of dying cells (teal dots and arrows).

**Fig. S3** No significant correlation between levels of aorta-specific cfDNA as measured using the aorta-specific DMRs and TUNEL staining in regions of normal WSS. Chr 11 DMR cfDNA levels (R^2^ 0.09, p = 0.32). Chr 18 DMR cfDNA levels (R^2^ 0.11, p = 0.36). Chr 20 DMR cfDNA levels (R^2^ 0.05, p = 0.61). Chr 22 DMR cfDNA levels (R^2^ 0.1, p = 0.29).

**Fig. S4** No significant correlation between the total levels of cfDNA and TUNEL staining in regions of elevated WSS (R^2^ 0.002, p = 0.89).
